# Burden of gastric cancer in Ecuador (2010–2021): a gender- and age-specific analysis using disability-adjusted life years (DALYs)

**DOI:** 10.3389/fepid.2025.1643323

**Published:** 2025-09-12

**Authors:** Ricardo Yajamín-Villamarín

**Affiliations:** ^1^ Pontifical Catholic University of Ecuador, Quito, Ecuador; ^2^ Value Health Economics Group-HEOR & HTA Consulting, Quito, Ecuador

**Keywords:** gastric cancer, disability-adjusted life years, Ecuador, disease burden, public health

## Abstract

**Background:**

Gastric cancer (GC) is a major public health issue and a leading cause of cancer-related mortality in Ecuador. Despite national cancer control efforts, the burden remains high, with variations by gender and age. This study aimed to quantify the burden of GC in Ecuador from 2010 to 2021 using Disability-Adjusted Life Years (DALYs), providing insights for public health strategies.

**Methods:**

A cross-sectional study was conducted using hospital discharge and mortality data from the National Institute of Statistics and Census (INEC). The study included all Ecuadorian individuals diagnosed with GC (ICD-10: C16) from 2010 to 2021. The burden of disease was estimated using DALYs, which combined Years of Life Lost (YLL) and Years Lived with Disability (YLD). Data were stratified by gender and age groups. Analyses were performed using Microsoft Excel and the DALY calculator in R v4.2.1.

**Results:**

Between 2010 and 2021, GC accounted for 802,135 DALYs in Ecuador, with an annual average of 66,845 DALYs. Males accounted for 57.2% of the total burden. The highest impact was observed in individuals aged 65–69 years. A progressive increase in disease burden was identified, particularly among older age groups.

**Conclusions:**

The findings highlight the need for targeted interventions, including early detection programs, risk reduction strategies, and improved healthcare access. Strengthening public health policies is crucial to mitigating the rising burden of GC in Ecuador.

## Introduction

Gastric cancer (GC) remains a significant global public health concern, ranking as the fifth most commonly diagnosed malignancy and the third leading cause of cancer-related mortality worldwide (1). In Ecuador, GC is a major contributor to cancer-related deaths, positioning the country 15th globally in incidence rates for both sexes. Between 2004 and 2015, GC accounted for 19,115 deaths in Ecuador, with 10,679 in men and 8,436 in women (2, 3). The burden of GC is particularly pronounced in Quito, the capital, where incidence and mortality rates mirror the national trend (4).

Multiple factors contribute to the high burden of GC in Ecuador. *Helicobacter pylori* infection is the most well-established risk factor, significantly increasing the likelihood of gastric carcinogenesis (5). Additionally, environmental factors, including soil contamination and exposure to carcinogenic compounds, have been identified as significant contributors to the increased incidence of GC in specific regions (6). Geographic variations in GC incidence and mortality suggest that altitude may also affect disease distribution. Research indicates that high-altitude regions, such as the provinces of Carchi and Cotopaxi, exhibit disproportionately high GC mortality rates. This pattern may be attributed to a combination of environmental exposures and genetic predisposition, highlighting the complex interplay between geographic, biological, and socioeconomic factors in shaping disease risk (3).

Socioeconomic disparities further exacerbate the burden of gastric cancer, as limited access to healthcare services and late-stage diagnoses contribute to poorer outcomes. Recent studies have demonstrated that patients with lower educational attainment and unskilled occupations have higher incidence and mortality rates for gastric cancer (7). Additionally, lack of health insurance is associated with delayed diagnoses and limited access to regular medical care, increasing the likelihood of detecting the disease at an advanced stage (8). These disparities in healthcare access and quality highlight the urgent need for public health interventions aimed at improving equity in gastric cancer care.

In response to this pressing health challenge, Ecuador has implemented various initiatives aimed at strengthening its cancer control strategies. In 2017, the Ministry of Public Health introduced a national cancer control plan focused on reducing cancer-related morbidity and mortality through early detection, improved diagnostic capabilities, and comprehensive patient management (4). Furthermore, initiatives such as the EquityCancer-LA project have been developed to enhance equity in access to early cancer diagnosis across Latin America. This initiative seeks to implement integrated care interventions, including the reinforcement of primary healthcare services, fast-track referral pathways, and patient navigation systems. A participatory approach, engaging healthcare professionals, policymakers, and patients, has been adopted to ensure that interventions are adapted to local contexts and effectively address existing barriers (9).

Despite these efforts, projections suggest that Ecuador may face a substantial increase in GC incidence in the coming decades. This anticipated rise highlights the critical need for targeted public health interventions, including strengthened screening programs, public awareness campaigns, and research focused on identifying and mitigating region-specific risk factors. A comprehensive, evidence-based approach is essential to reducing the burden of GC and improving health outcomes for affected populations in Ecuador (10).

While mortality and incidence data provide valuable information on the magnitude of GC, they do not capture the full impact of the disease on population health, particularly regarding years lived with disability. Disability-adjusted life years (DALYs) offer a more comprehensive measure by combining both premature mortality and morbidity, reflecting the total health loss attributable to a given condition (11). In countries like Ecuador, where holistic assessments of cancer burden remain limited, estimating DALYs helps address a critical gap in public health data by providing insights not only into fatal outcomes but also into the non-fatal health consequences of GC. This approach can support more effective health policy design and resource allocation decisions aimed at reducing the burden of disease (12, 13).

This study aims to use Ecuadorian data and the methodology established by the World Health Organization (WHO) to estimate the country's disease burden from gastric cancer, measured in DALYs. By quantifying the impact of GC in terms of DALYs, this research seeks to provide valuable insights for public health decision-making and resource allocation.

## Methods

### Data

This study conducted a comprehensive analysis of the burden of stomach cancer in Ecuador over a 12-year period, from 2010 to 2021. The primary data source was the statistical record of hospital beds and discharges provided by the National Institute of Statistics and Census (INEC), which serves as Ecuador's central repository for hospital-based health data. This consolidated database integrates information from both public and private healthcare institutions, ensuring a comprehensive representation of the national population (14).

Data extraction was performed using the International Statistical Classification of Diseases and Related Health Problems, 10th Revision (ICD-10). Specifically, code C16 was employed to identify cases of malignant neoplasms of the stomach. These records underwent systematic processing, cleaning, and structuring in Microsoft Excel to maintain data integrity. The final analysis was conducted using the Disability-Adjusted Life Year (DALY) calculator in R v4.2.1, adhering to standardized burden of disease methodologies (15).

The study population comprised all Ecuadorian individuals diagnosed with stomach cancer within the study period. For a more granular epidemiological assessment, data were stratified by sex and age groups, categorized in five-year intervals. This stratification facilitated a detailed examination of disease patterns, enabling comparisons across demographic subgroups and providing a clearer understanding of the burden of stomach cancer in different population segments. By employing a rigorous and structured methodological approach, this study aimed to generate robust evidence on the national burden of stomach cancer, offering valuable insights for the development of targeted public health interventions and the optimization of healthcare resource allocation.

### Burden of disease estimation for stomach cancer in Ecuador (2010–2021)

The burden of stomach cancer in Ecuador was estimated using the DALYs metric, which quantifies the overall impact of the disease by combining Years of Life Lost (YLL) due to premature mortality and Years Lived with Disability (YLD). This methodology follows the guidelines proposed by the WHO to ensure comparability across global burden of disease studies (16).

### Years of life lost (YLL)

YLL was calculated based on the number of deaths attributed to stomach cancer (ICD-10: C16) during the study period (2010–2021) and the residual life expectancy at the age of death. The estimation followed standardized life expectancy values, assuming a life expectancy at birth of 86 years for both sexes, in accordance with WHO recommendations. Specific life expectancy values were applied for each five-year age group to maintain consistency with standard burden of disease methodologies. No time discounting or age weighting was applied in the YLL calculation to facilitate international comparability (16).

### Years lived with disability (YLD)

YLD was estimated to account for the non-fatal burden of stomach cancer. The calculation was based on disease prevalence rather than incidence, in line with the methodology used in Global Burden of Disease (GBD) studies. The estimation considered the number of individuals diagnosed with stomach cancer and recorded in hospital discharge data during the study period. Disability weights were derived from the GBD study, which provides standardized values on a scale from 0 (perfect health) to 1 (equivalent to death) (17).

To refine the estimation, disease severity was considered by classifying cases into different health states, ranging from early-stage stomach cancer with mild symptoms to advanced-stage disease with significant impairment. The YLD calculation also accounted for variations by sex and age group, categorizing the population into five-year intervals. Since this study was based on prevalence data, the duration of the disease was included to more accurately reflect its long-term burden on affected individuals.

### Calculation of DALYs

The total DALYs were computed as the sum of YLL and YLD for each age and sex category. The final burden of disease estimates was stratified by age and sex to facilitate a more detailed evaluation of the impact of stomach cancer across Ecuador.

Data processing was conducted using Microsoft Excel for data management and cleaning, while DALY computations were performed using the DALY calculator in R v4.2.1, following the standardized burden of disease methodologies (15). The analysis adheres to established guidelines to ensure methodological consistency and comparability with global and regional burden of disease studies.

This assessment provides a comprehensive quantification of the disease burden attributable to stomach cancer in Ecuador. The findings offer critical insights to inform public health policies, optimize resource allocation, and develop targeted interventions aimed at reducing mortality and improving the quality of life of affected individuals.

## Results

### Burden of disease estimation for stomach cancer in Ecuador (2010–2021)

The total burden of stomach cancer in Ecuador was estimated at 802,135 years for the period 2010–2021, with an annual average of 66,845 DALYs. This metric captures both premature mortality and the years lived with disability, providing a comprehensive measure of the disease's impact.

When disaggregated by sex, male individuals accounted for 458,309 DALYs, representing 57.2% of the total burden, with an annual average of 38,192 DALYs. Meanwhile, female individuals contributed 343,826 DALYs, representing 42.8% of the total burden, with an annual average of 28,652 DALYs (Table 1). These findings suggest that stomach cancer has a higher overall impact on the male population in Ecuador, a pattern consistent with global epidemiological trends.

**Table 1:** Burden of disease estimation of stomach cancer (ICD-10: C16) in Ecuador from 2010 to 2021.

Year	YLL		YLD		DALYs	
	Male	Female	Male	Female	Male	Female
15–19	2,129	1,404	16	13	2,144	1,417
20–24	5,125	3,346	35	30	5,160	3,375
25–29	9,091	6,408	62	55	9,153	64,63
30–34	14,270	12,645	91	104	14,362	12,750
35–39	17,292	18,236	107	142	17,399	18,378
40–44	24,161	21,976	151	176	24,312	22,151
45–49	30,796	27,351	219	239	31,015	27,590
50–54	40,915	31,347	332	265	41,247	31,612
55–59	49,840	31,838	431	288	50,271	32,126
60–64	53,171	33,932	503	317	53,674	34,249
65–69	55,576	35,206	600	365	56,176	35,571
70–74	53,351	34,503	678	401	54,029	34,903
75–79	43,231	30,989	677	393	43,908	31,382
80–84	29,808	24,863	634	365	30,442	25,227
85+	24,455	26,176	562	454	25,017	26,630
Total	453,211	340,219	5,098	3,607	458,309	343,826

YLL (Years of Life Lost) represents the years of life lost due to premature mortality. YLD (Years Lived with Disability) corresponds to the years lived with disability. DALYs (Disability-Adjusted Life Years) are the sum of YLL and YLD, indicating the total burden of disease.

### Trends in disease burden by age group (2017–2021)

**Figure 1:**
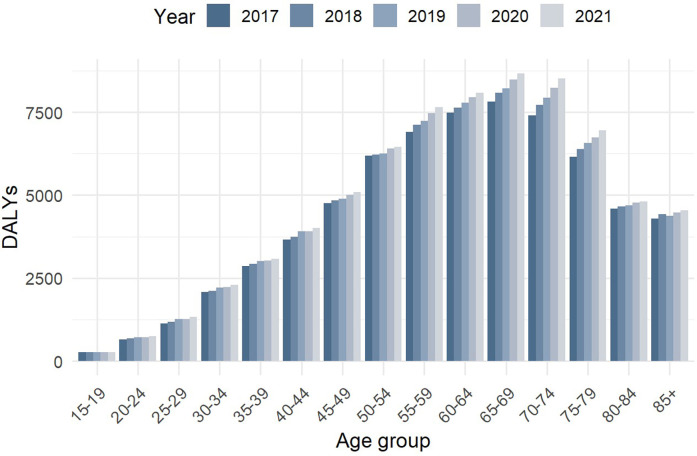
Evolution of burden of disease (DALYs) by age groups from 2017 to 2021.

Figure 1 illustrates the evolution of DALYs due to stomach cancer across different age groups from 2017 to 2021. A progressive increase in DALY values was observed, indicating a rising burden of disease over this period.

The age group most affected was 65–69 years, particularly in 2021, when the highest DALY values were recorded. This suggests that individuals in this age range experienced a disproportionately higher impact of stomach cancer, likely due to increasing incidence, disease progression, and associated comorbidities.

Moreover, the burden was notably higher in individuals aged 55–79 years, reaffirming that stomach cancer primarily affects older adults. In contrast, younger age groups (below 45 years) exhibited significantly lower DALY values, suggesting that stomach cancer remains relatively rare among younger populations but still contributes to premature mortality in certain cases.

These findings emphasize the age-dependent nature of stomach cancer, where older adults, particularly males, are at higher risk of mortality and disability due to the disease. Understanding these trends is essential for targeted prevention strategies, early detection programs, and healthcare resource allocation in Ecuador.

## Discussion

This study has assessed the burden of stomach cancer in Ecuador, analyzing gender and age group differences from 2010 to 2021, using DALYs as a comprehensive measure to quantify the impact of this disease on the population. The results reveal significant patterns that warrant detailed analysis and considerations for the development of effective public health strategies.

### Gender differences in the burden of stomach cancer

Our findings indicate that males consistently experience a higher burden of stomach cancer, measured in DALYs, compared to females across all age groups in Ecuador. This disparity may be attributed to several factors, including gender-specific risk behaviors, occupational exposures, and biological differences. Previous studies have demonstrated that males have a higher risk of developing stomach cancer than females, potentially due to the influence of androgen receptors on gastric carcinogenesis (18). Additionally, global data suggest that the incidence of stomach cancer is 1.5–3 times higher in males than in females (19).

Beyond biological factors, social and behavioral determinants likely play a substantial role in explaining these gender disparities. Men in Ecuador are more frequently exposed to established risk factors such as smoking, alcohol consumption, and dietary habits characterized by high salt intake (20, 21). Moreover, Helicobacter pylori infection is more prevalent among males, which may be linked not only to biological susceptibility but also to differences in healthcare-seeking behavior and delayed diagnosis. These patterns mirror trends observed in other Latin American countries and globally, where men consistently show higher incidence and mortality rates for gastric cancer (19, 22). Recognizing these multifactorial causes is essential to developing targeted and equitable prevention and treatment interventions.

### Age distribution of stomach cancer burden

Analysis by age groups reveals that the burden of stomach cancer increases with age, reaching its peak in individuals aged 65–69 years. This finding aligns with international studies showing an increased incidence of stomach cancer in older adults (23). Identifying this age group as particularly vulnerable highlights the need for early detection programs and timely treatment strategies to improve prognosis and quality of life for affected patients.

As with gender differences, contextualizing the age-related patterns within Ecuador's social and healthcare landscape is important. Delays in diagnosis among older adults may be exacerbated by barriers to accessing specialized medical services, especially in rural or underserved regions (24). Comparisons with regional and global data suggest that Ecuador's pattern of increasing gastric cancer burden with age is consistent with trends observed in both high- and middle-income countries, further emphasizing the universal nature of this public health challenge (22, 25).

### Comparison of total burden between genders

The results indicate that males experience a higher overall burden of stomach cancer in the Ecuadorian population during the analyzed period. While the reasons behind this disparity are multifactorial, contributing factors may include differential exposure to carcinogenic agents, dietary habits, and a higher prevalence of Helicobacter pylori infections among males. Studies have suggested that males have a greater predisposition to developing stomach cancer due to hormonal and genetic factors (18).

Incorporating both social determinants and biological predispositions provides a more comprehensive understanding of these disparities. Differences in occupational exposure to carcinogenic substances, health-related behaviors, and cultural factors influencing healthcare utilization contribute to a gender gap that is not unique to Ecuador but is observed in many countries with similar epidemiological profiles (19, 22).

### Public health implications

The findings of this study, which analyzed the burden of gastric cancer in Ecuador from 2010 to 2021, have significant public health implications. The higher burden of this disease, observed in males and older age groups, suggests the need for targeted prevention and control strategies to effectively address these disparities.

The male population exhibits a significantly higher burden of gastric cancer, likely due to a combination of behavioral and biological factors. Smoking and alcohol consumption have been identified as major risk factors for gastric cancer, and these behaviors are more prevalent among men, particularly in regions with high rates of the disease (20). A meta-analysis of cohort studies determined that current smokers have a 62% higher risk of developing gastric cancer compared to non-smokers, with an even stronger association for cancers located in the gastric cardia (20). Additionally, high dietary salt intake has been shown to be a significant risk factor for gastric cancer, acting synergistically with *Helicobacter pylori* infection and promoting abnormal cell proliferation (26). Public health campaigns targeting these risk factors could significantly reduce the incidence of gastric cancer, particularly among men.

Ecuador's aging population also demands proactive cancer care strategies, especially as the burden of gastric cancer increases significantly with age. This study aligns with global findings showing a higher incidence of the disease in individuals aged 65 and older (27). Early detection programs, such as endoscopic screening for at-risk populations, could be crucial in identifying gastric cancer at early stages, ultimately improving survival outcomes (28). A study in Japan reported a 30% reduction in gastric cancer mortality in populations that underwent endoscopic screening, highlighting the effectiveness of this strategy (28). Furthermore, advancements in endoscopic techniques, such as endoscopic submucosal dissection, have shown favorable long-term outcomes in treating premalignant gastric lesions and early-stage cancers (29).

Ensuring equitable access to healthcare services in both urban and rural settings is also essential. Healthcare access disparities are well-documented in Ecuador and other Latin American countries, exacerbating the burden of gastric cancer in underserved populations (30). A study on healthcare access barriers in rural southern Ecuador identified financial, structural, and cognitive obstacles that hinder access to adequate medical care (30). Addressing these disparities by expanding diagnostic and treatment services in rural areas is a crucial step toward reducing the disease burden.

Finally, future research should explore the genetic and environmental interactions that contribute to the higher burden of gastric cancer in men. A deeper understanding of these factors will enable the development of more personalized prevention strategies tailored to the specific needs of different population subgroups. Additionally, studies on the effectiveness of screening and early detection programs could provide valuable insights for refining public health interventions, ensuring they are both effective and sustainable in reducing the burden of gastric cancer.

## Conclusion

This study provides a detailed overview of the burden of stomach cancer in Ecuador, highlighting significant differences between genders and age groups. Understanding these patterns is essential for designing more effective and equitable public health interventions aimed at reducing the incidence and impact of this disease on the Ecuadorian population.

### Limitations

Despite efforts to address the burden of stomach cancer in Ecuador in this study, several limitations must be acknowledged. The quality of the data used relies heavily on the accuracy and completeness of national cancer records, which may be affected by underreporting, subregistration, and potential misclassification, particularly in rural or underserved areas. Additionally, the estimation of years lived with disability (YLD) was based on hospital-based prevalence data, which likely underestimates the true burden among populations with limited access to healthcare services. The study used nationally aggregated data from 2010 to 2021, without conducting subnational analyses by province or region.

Furthermore, the results do not include statistical significance testing, which limits the robustness and inferential capacity of the findings. The study also does not incorporate predictive modeling or trend analysis to quantify future projections of gastric cancer burden, despite recognizing the potential for an increase in incidence. Another limitation is the absence of intersectional analyses that consider variables such as educational level, ethnicity, or area of residence, which would provide a more comprehensive understanding of inequities.

## Data Availability

The original contributions presented in the study are included in the article/Supplementary Material, further inquiries can be directed to the corresponding author.
